# Bulk high-entropy nitrides and carbonitrides

**DOI:** 10.1038/s41598-020-78175-8

**Published:** 2020-12-04

**Authors:** Olivia F. Dippo, Neda Mesgarzadeh, Tyler J. Harrington, Grant D. Schrader, Kenneth S. Vecchio

**Affiliations:** 1 Materials Science and Engineering Program, UC San Diego, La Jolla, CA 92037 USA; 2 Department of NanoEngineering, UC San Diego, La Jolla, CA 92037 USA

**Keywords:** Materials science, Structural materials, Ceramics

## Abstract

High-entropy ceramics have potential to improve the mechanical properties and high-temperature stability over traditional ceramics, and high entropy nitrides and carbonitrides (HENs and HECNs) are particularly attractive for high temperature and high hardness applications. The synthesis of 5 bulk HENs and 4 bulk HECNs forming single-phase materials is reported herein among 11 samples prepared. The hardness of HENs and HECNs increased by an average of 22% and 39%, respectively, over the rule-of-mixtures average of their monocarbide and mononitride precursors. Similarly, elastic modulus values increased by an average of 17% in nitrides and 31% in carbonitrides over their rule-of-mixtures values. The enhancement in mechanical properties is tied to an increase in the configurational entropy and a decrease in the valence electron concentration, providing parameters for tuning mechanical properties of high-entropy ceramics.

## Introduction

High entropy ceramics have been gaining traction in recent years^1,2^, since Rost et al. adapted the idea of high-entropy alloys^3,4^ to synthesize the first entropy-stabilized oxide^5^. The field of bulk high-entropy ceramics has grown to include borides^6^, carbides^7–11^, silicides^12,13^, perovskite oxides^14^, and fluorite oxides^15^. Many of these bulk high-entropy ceramics have increased hardness over their constituents^6,7,9^, and have the potential for increased phase stability at high temperatures^16^, according to the increased entropy term (S) in the Gibb’s free energy equation G = H-TS.

Transition metal nitrides and carbonitrides are used for their high hardness, wear resistance, and refractory character—having melting temperatures over 1800 °C^17^, with some having melting temperatures above 4000 °C^18^. High-entropy versions of these materials show potential to augment their already high hardness and thermal stability, making them promising candidates for leading edges and thermal protective components in aerospace applications, which require bulk materials^19^. HENs have been synthesized as a powder^20^ and as thin films^21–36^; HECNs have also been synthesized as thin films^37,38^. High-entropy nitrides (HENs) and high-entropy carbonitrides (HECNs) have not been synthesized in bulk form until recently, when a paper by Wen et al*.* reported the synthesis of one bulk HEN composition and one bulk HECN composition, (HfNbTaTiZr)N and (HfNbTaTiZr)CN, respectively^39^. In addition to these compositions, we report the first bulk synthesis of 4 other HECN compositions and 3 other HEN compositions.

HENs and HECNs in particular offer interesting opportunities for tuning mechanical properties over existing bulk high-entropy ceramics, the first of which is their configurational entropy. Higher configurational entropy is associated with favorable “high-entropy” material behavior, such as increased strength and phase stability. Methods for calculating ideal configurational entropy of high-entropy ceramic compositions will be discussed in this paper, which indicate that the configurational entropy of a carbonitride is significantly higher than that of a nitride or carbide. The ideal configurational entropy for an equiatomic and stoichiometric five-metal nitride is 0.8R, and for a carbonitride is 1.15R, where R is the gas constant. This entropy increase upon addition of a second anion to a high-entropy ceramic system is equal to that of adding five additional metal species, giving carbonitrides a major advantage in entropy-related properties.

In addition to entropy, valence electron concentration (VEC) provides an opportunity for tuning mechanical properties, where HENs have the advantage of a higher VEC. Transition metal nitrides, carbides, and carbonitrides have mixed bond character, considered partially covalent, ionic, and metallic. This gives rise to many of the interesting properties of these materials, for example maintaining the high melting temperatures of ionic/covalently bonded materials and the high thermal and electrical conductivity of metallic materials^17^. Changing the composition of the HEN or HECN can shift the bond character, which can be quantified using the material’s VEC—calculated as the number of valence electrons per formula unit. VEC can be manipulated by varying the metals contained in the ceramic, and in addition, nitrogen has one more valence electron than carbon, giving HECNs and HENs higher VECs than high-entropy carbides. Increasing VEC in cubic carbides, nitrides, and carbonitrides decreases hardness^9,40^ and increases toughness^41^. Therefore, HENs, having higher nitrogen content and thus higher VEC than carbon-containing high-entropy ceramics, should display lower hardness, thus indicating potential for future synthesis of more ductile ceramics.

The aim of this study is twofold: (i) to report the first synthesis of five bulk HENs and six bulk HECNs, and (ii) to elucidate the effect of configurational entropy and VEC on the mechanical properties of bulk high-entropy ceramics. Consistent with the current literature on bulk high-entropy ceramics, the single-phase character of all HENs and HECNs will be analyzed. A single-phase high-entropy material is defined as having one majority crystal structure and a homogeneous distribution of elements within the material; conditions chosen to ensure that randomness, and therefore configurational entropy, is maximized. Bulk high-entropy ceramics are often equiatomic, i.e. all elements are in equal atomic proportions, though this is not a requirement to categorize a high-entropy ceramic as single-phase. In this work, single-phase or multi-phase character of these novel HENs and HECNs is experimentally determined for all 11 samples through XRD and EDS. Additionally, nanoindentation hardness and elastic modulus data are presented, in comparison with expected rule of mixtures (ROM) averages of the constituents of each composition. Mechanical properties are also analyzed in terms of configurational entropy and VEC, defining trends that may influence future design of ceramic materials.

## Materials and methods

### Synthesis

Precursor powders of graphite, CrN, CrC, HfC, Mo_2_C, NbN, NbC, TaN TaC, TiN, TiC, VC, W_2_C, ZrN, and ZrC were procured from Alfa Aesar (> 99% purity); HfN was procured from Reade Advanced Materials (99.5% purity); VN was procured from American Elements (99% purity).

For nitride and carbonitride compositions listed as HENs #1–5 and HECNs #1–6 respectively, appropriate amounts of precursor powders were hand-mixed, to achieve an equiatomic mixture, calculated on the metals basis. An additional 5 atomic percent graphite powder was added to all of the samples, to aid in sintering and reduce oxides present in the precursor powders. To achieve particle size reduction and improved blending, hand-mixed powders were then high-energy ball milled using a SPEX 8000D mill (SpexCertPrep, NJ, USA) for three hours total, in increments of 30 min with 10-min cool-down periods in between. Tungsten carbide milling media was used (ball to powder ratio 2:1), and stearic acid was added as a lubricant to the milling process. The SPEX milling and subsequent powder handling was done in an argon glove box to prevent oxidation.

Powders were then densified using spark plasma sintering (SPS, Thermal Technologies, CA, USA) under vacuum (below 30 mtorr) using a temperature ramp rate of 100 °C/min and held at temperature for 1 h under a pressure of 50 MPa. During heating, whenever significant sample off-gassing occurred, heating was paused to allow the vacuum level to stay below 30 mtorr. Chromium containing compositions were sintered at a maximum temperature of 1600 °C, and compositions that did not contain chromium were sintered at 2200 °C. Mononitrides (defined as containing one transition metal, for example CrN) were each synthesized in the SPS at approximately 300 °C below their respective melting temperatures. Samples were sintered in 20-mm graphite dies lined with graphite foil. The outside surfaces of sintered samples were ground away using an 80-grit diamond grinding disc, to remove residual carbon contamination before characterization.

### Characterization

Crystal structures of sintered samples were characterized using powder X-ray diffraction (XRD) in a Rigaku Miniflex diffractometer with Cu K-α radiation. Microstructure and metal content were characterized in a scanning electron microscope (SEM) (Apreo, Thermo-Fisher) with energy dispersive X-ray spectroscopy (EDS) (Oxford Instruments). Densities were measured using Archimedes method; chemical analysis of carbon and nitrogen content were characterized using a Perkin Elmer 2400 Series II CHNS/O Elemental Analyzer (CHNS).

### Mechanical testing

Bulk, sintered samples were first ground and polished using diamond media, with an 0.04 µm colloidal silica suspension final polish. Nanoindentation according to the Oliver and Pharr method^42^ was performed using 300 mN force to obtain asymptotic hardness, avoiding indentation size effects, which are present below 100 mN, as shown in Supplementary Fig. S2. Nanoindentation was performed with a diamond Berkovich indenter in accordance with the ISO 14577 standard. On each sample, a grid of 100 indents were done, spaced 30 µm apart, and the typical indentation depth was approximately 800 nm. Outliers were removed by removing data points with an indentation depth greater than 1400 nm, which were confirmed by optical microscopy to have intersected pores near the surface. The maximum and minimum 5% of data points were removed before averaging the values. Removing outliers in this fashion was necessary for the mononitrides, which had lower sintered densities than the high-entropy compositions, causing more outliers in the low end of the data and artificially lowering the average nanoindentation results. The nanoindentation data for monocarbides is published in another work by the authors, using comparable synthesis methods and identical indentation parameters^9^. The rule of mixtures (ROM) values for modulus and hardness comparison were taken as a weighted average value of the mononitride and monocarbide compounds, based on the measured composition of each high-entropy sample. Poisson’s ratios for all precursor compounds were gathered from the literature (see Table 2), and the average value was used for each high entropy composition. The Poisson’s ratios for the high entropy compositions were all within the range of 0.24–0.29.

## Results

### Single-phase formation: crystal structure

**Figure 1:**
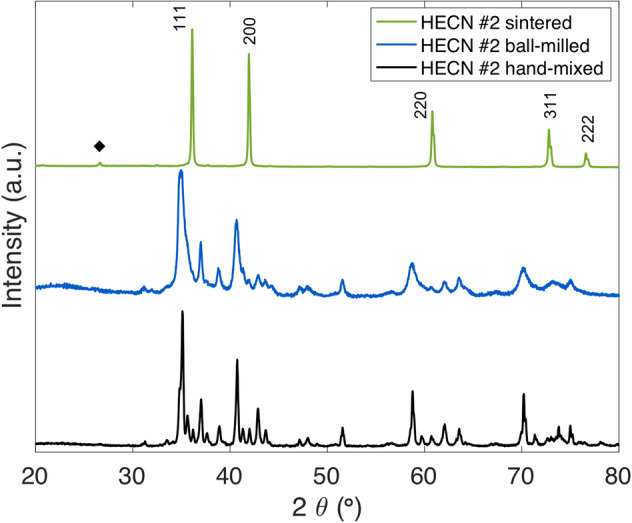
Example progression of HECN #2 (CrNbTaTiV) (CN) through the processing steps: hand-mixed (bottom), high-energy ball-milled (middle), and then sintered (top). A residual graphite peak is marked with a black diamond. The FCC-rocksalt phase is marked with Miller indices.

XRD results in Fig. 1 show the phase consolidation of the precursor mononitrides and monocarbides into a single FCC phase throughout the processing steps: hand-mixing, ball-milling, and SPS densification, even though many of the precursor compositions do not form a room temperature stable FCC-rocksalt phase on their own and/or have many room-temperature polymorphs. A list of phases present in the precursor powders is provided in Supplementary Table S1.

**Figure 2:**
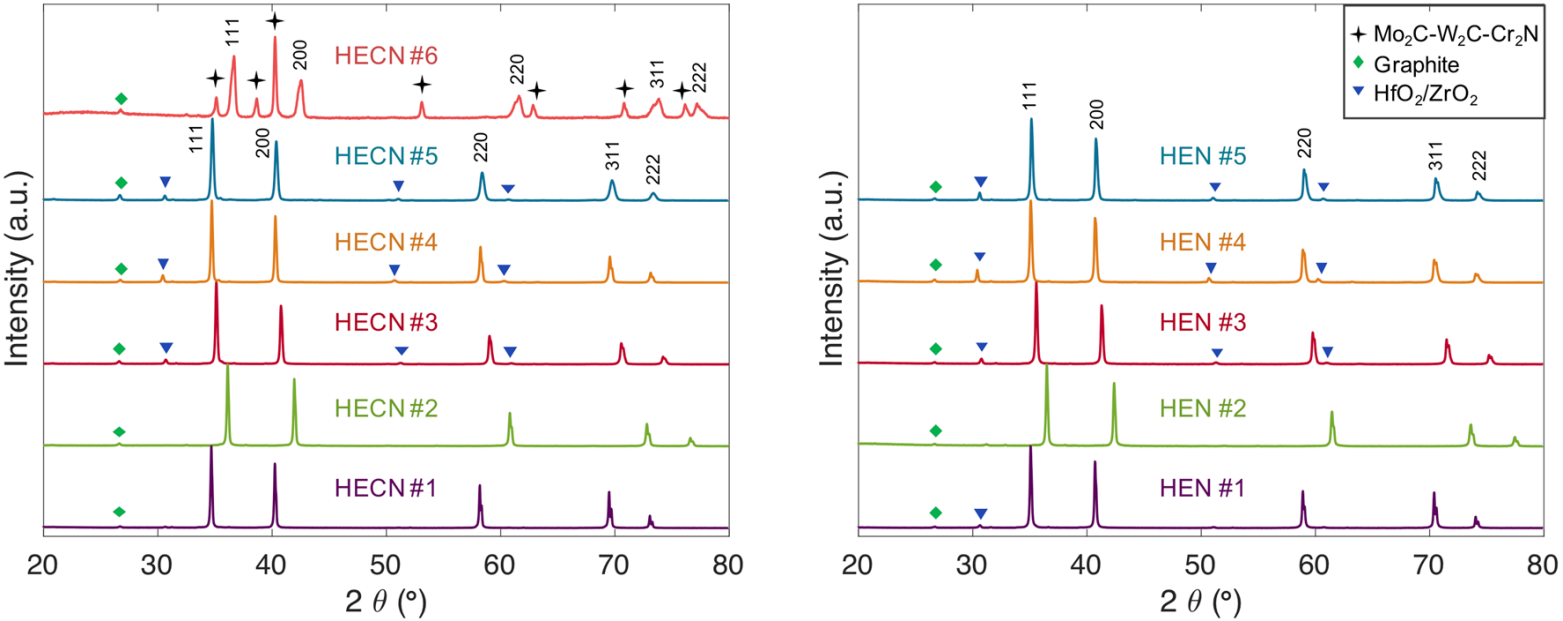
XRD of all 6 carbonitride (left) and 5 nitride (right) compositions. HEN and HECN #1–5 are FCC-rocksalt structures, with some residual native oxides present. HECN #6 shows multiple phases. Intensities are normalized by the highest intensity present in each spectrum.

After sintering, HEN and HECN samples #1–5 all show a majority FCC-rocksalt phase (F m − 3 m), as shown in Fig. 2. HECN #6 is the only material synthesized with two major crystal structures; it contains an FCC phase and a hexagonal phase. Minor hafnium/zirconium oxide peaks are visible in some XRD patterns; these oxides are native to the precursor powders. For a complete list of all minor oxide phases identified in each material, see Supplementary Table S2. Minor graphite peaks are due to the 5 atomic percent graphite powder added as sintering aid and to help reduce native oxides.

### Single-phase formation: random distribution of elements

**Figure 3:**
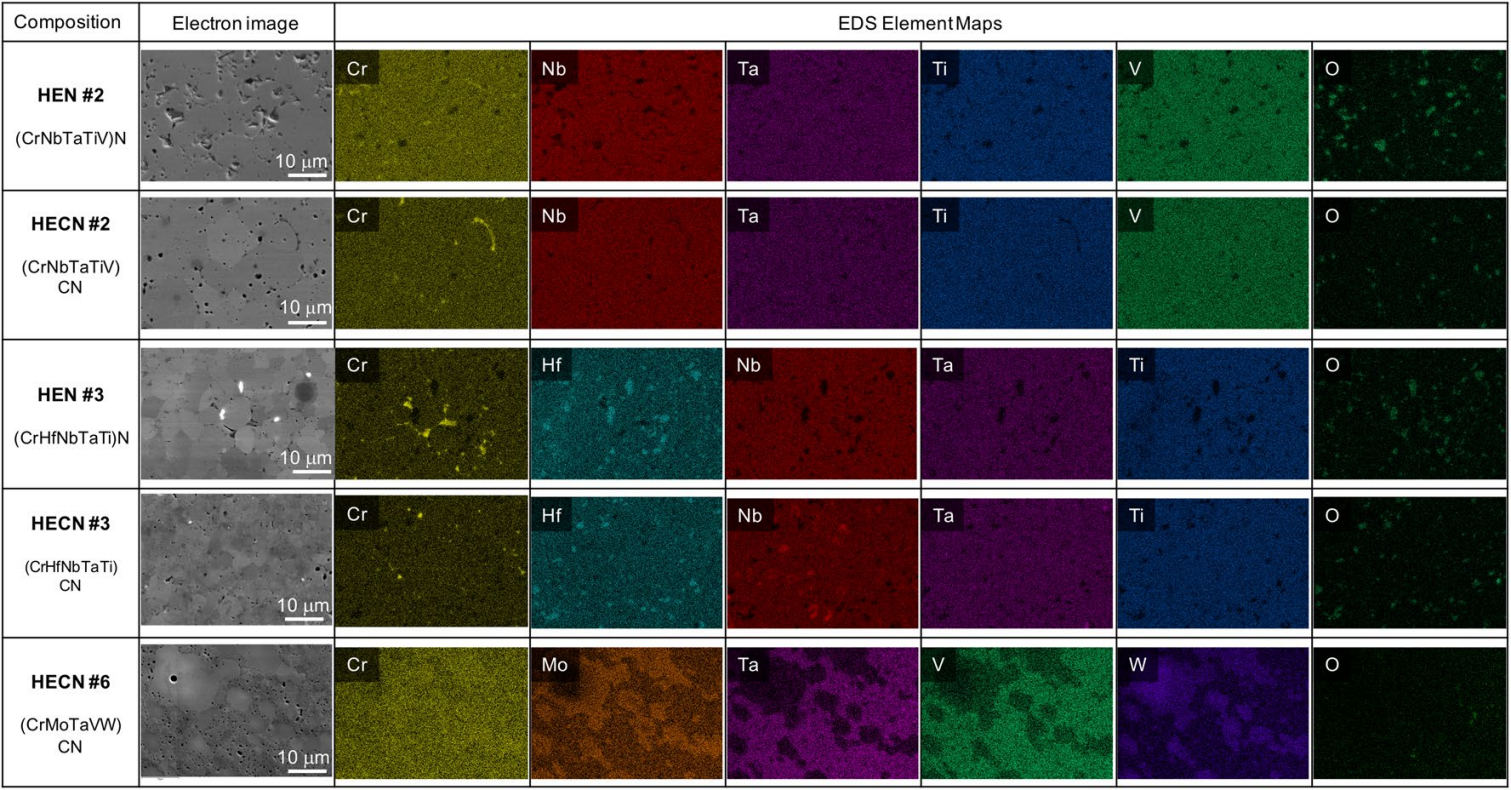
Secondary electron images and corresponding elemental EDS maps of selected compositions. HEN #2, HECN #2, and HEN #3 have elements homogenously distributed throughout the matrix, and thus are considered single-phase high-entropy ceramics. HECN #3 contains islands of segregated niobium, thus it does not have the compositional homogeneity required to be single-phase. HECN #6 is a dual phase ceramic, with clear elemental segregation. Oxygen maps are included to indicate the elemental segregation of minor native oxide phases (such as Cr_2_O_3_ and HfO_2_), separate from the high-entropy matrix phases.

To maximize ideal configurational entropy, the sample should be a homogenous mixture, with the elements in equal atomic proportions on each sublattice. First, the homogeneity of the samples will be discussed. EDS maps showing chemical homogeneity of selected compositions are shown in Fig. 3 (EDS maps of all compositions are shown in Supplementary Fig. S1); there is a small variation in chemical homogeneity from sample to sample. For example, HEN #2 and HECN #2 have good chemical homogeneity, meaning the elements are randomly distributed, except for a minor chromium oxide phase. Oxygen maps are included to help the reader distinguish which chemical inhomogeneities are due to minor oxide phases, and a list of oxide phases present in each sample is included in Supplementary Table S2. The primary matrix structure is close to equiatomic throughout the samples. Both HEN #3 and HECN #3 contain minor amounts of chromium oxide and hafnium oxide, but otherwise the elements in the matrix are homogenously distributed. In HECN #3, however, there are additional islands of increased niobium content of about 2 atomic % higher than other regions. Therefore, HECN #3 is not considered to have good compositional homogeneity, and therefore is not truly single-phase, even though there is only one FCC crystal structure present in XRD.

To understand why some high-entropy ceramic compositions form a compositionally homogenous single phase and some do not, the Entropy Forming Ability (EFA) descriptor concept can be employed. Sarker et al*.* created this descriptor to predict how readily specific compositions would form homogenous single-phase high entropy carbides^10^. The EFA ratings imply that there is a continuum of how easy it is to synthesize a high-entropy ceramic phase with one crystal structure and homogeneous random mixing of elements. Indeed, it was shown by Kaufmann et al*.* that high-entropy carbides with lower EFA values, displaying some chemical segregation (but only one detectable crystal structure), could be homogenized with a longer annealing time^11^. Similarly, HECN #3 can be considered to be in the middle of the EFA continuum, where it could potentially be synthesized as a homogenous single-phase, but would require more energetic input (i.e. mechanical milling, or time at temperature) during synthesis. Though it is possible to homogenize chemical segregation islands in this way, the differences are pointed out here using identical synthesis processes as these differences may indicate the ease of forming single phase compounds of these compositions, which is an indication of their entropic stability.

As shown in Fig. 3, HECN #6 demonstrates the most extreme case of chemical segregation, which is not surprising given that two majority crystal structures are seen in XRD. HECN #6 shows clear segregation of elements into two distinct phases. To understand the segregation behavior in HECN #6, the slight difference in synthesis technique for this sample must be noted. Due to limitations in availability of mononitrides, this sample was made using VN, CrN, TaN, Mo_2_C, W_2_C, and graphite. All other carbonitride samples were made by mixing both the monocarbides and mononitrides of each metal (i.e. the (CrMoTaVW)(CN) sample would have been made with CrN, CrC, MoN, MoC, TaN, TaC, VN, VC, WN, and WC if the precursors were available). Therefore, it is not surprising that the Mo and W cluster together in EDS, matching the hexagonal Mo_2_C-W_2_C phase present in XRD. Interestingly, the chromium content is relatively homogenous, due to the Cr_2_N phase present in the chromium nitride precursor powder, which mixes favorably with the Mo_2_C-W_2_C phase. One of the biggest challenges in synthesis of bulk HENs and HECNs is the lack of readily available mononitride powders.

### Equiatomic phase formation

The composition of each sample in atomic percent can be found in Table 1, with metal data from EDS and light element (C, N) data from CHNS combustion analysis. The precursor powders are mixed in equiatomic proportions, but there are some variances in the final sintered compositions. Nitrogen content is consistently lower than equiatomic (50 at% for the nitrides and 25 at% for the carbonitrides), which is consistent with knowledge that nitrogen vacancies are prevalent in nitrides at high temperatures^17,43^. More surprisingly, in some compositions, the chromium content is significantly lower than equiatomic throughout the sample.

**Table 1:** Composition of experimental samples in atomic percent, calculated from EDS analysis of metal content and CHNS chemical analysis of carbon and nitrogen content. Metals are expected to be in equiatomic proportion.

Atomic %	C	N	Cr	Hf	Nb	Ta	Ti	V	Zr	Mo	W	C + N subtotal	Metals subtotal
HEN #1	9.9	36.7	–	10.9	10.8	10.5	10.6	–	10.8	–	–	46.5	53.6
HEN #2	4.0	41.5	12.0	–	11.0	10.5	10.4	10.7	–	–	–	45.4	54.6
HEN #3	5.9	41.4	6.4	12.0	11.8	10.8	11.6	–	–	–	–	47.3	52.6
HEN #4	6.5	41.2	4.0	11.8	12.2	–	12.6	–	11.6	–	–	47.7	52.2
HEN #5	6.0	41.9	4.7	12.1	–	11.3	12.2	–	11.7	–	–	47.9	52.0
HECN #1	30.0	17.6	–	10.0	10.9	10.4	10.5	–	10.7	–	–	47.5	52.5
HECN #2	26.4	18.5	10.3	–	11.1	10.7	11.7	11.4	–	–	–	44.9	55.2
HECN #3	27.8	19.8	5.4	12.2	11.8	11.7	11.3	–	–	–	–	47.6	52.4
HECN #4	27.7	19.8	2.7	12.4	12.9	0	12.2	–	12.4	–	–	47.4	52.6
HECN #5	29.1	18.0	3.7	12.2	–	12.1	12.5	–	12.4	–	–	47.1	52.9
HECN #6	32.1	7.4	12.1	–	–	12.1	0.0	11.8	–	12.3	12.2	39.5	60.5

All chromium-containing compounds were sintered under the same conditions: 1600 °C under vacuum for 1 h, as detailed in the Methods section of this work. If the high-entropy composition is not stable at this temperature, chromium will tend to evaporate. This can happen via one of two pathways: carbothermal reduction of Cr_2_O_3_ to form chromium metal and carbon monoxide gas, and then chromium metal vaporization, which occurs at temperatures above 825 °C under vacuum^44^, or significant nitrogen evaporation in chromium nitride and subsequent chromium metal evaporation. Chromium is the most sensitive element because chromium nitride and chromium carbide have the lowest melting temperatures of all the precursors.

Atomic proportion of elements does not affect whether a high-entropy ceramic is considered single-phase, but it can act as an indicator of high-temperature stability of the compound. If the high-entropy ceramic phase is stable at high temperatures, it will tend to maintain its equiatomic composition, as in HEN #2 and HECN #2, which did not lose chromium content during synthesis, i.e. maintained equiatomic character. This may be used as an indicator of high-temperature phase stability, and is also a characteristic of having a higher EFA value, as Kaufmann et al*.* noted chromium loss in lower EFA carbides^11^. High-temperature phase stability and thus increased melting temperatures are theorized to be an asset of high-entropy systems, due to the increased contribution of entropy (S) to the Gibb’s free energy G = H-TS at high temperatures^16^. However, it should be noted that we have not determined that these HENs and HECNs are entropy-stabilized, as carbides and nitrides have some mutual solubility^17^ as well as strong enthalpic contributions to phase stability^10^.

### Mechanical properties

Nanoindentation was performed on all ten of the FCC-structured compositions, i.e. HENs #1–5 and HECNs #1–5. In order to compare the mechanical properties of the high-entropy nitrides and carbonitrides to the properties of their constituents, each monocarbide and mononitride was also synthesized in a similar fashion and mechanical testing was performed under the same conditions. Most of the monocarbides were synthesized and tested in another work by the authors^9^. The results of the mononitrides and chromium carbide are shown in Table 2. Most of the data for nanoindentation hardness and elastic modulus for transition-metal mononitrides is not otherwise available in the literature for comparison.

**Table 2:** Nanoindentation elastic modulus, hardness, and Poisson’s ratios for every monocarbide and mononitride used as a precursor for the HENs and HECNs. Modulus and hardness are measured from samples synthesized via SPS. Poisson’s ratios are gathered from the literature, and mechanical properties for carbides (except CrC) are from Ref.^9^.

Composition	Nanoindentation modulus (GPa)	Nanoindentation hardness (GPa)	Poisson’s ratio	References
TiN	532 ± 25	21.1 ± 2.7	0.22	^45,46^
TiC	489	31	0.19	^9,47^
ZrN	441 ± 23	18.6 ± 1.6	0.26	^46^
ZrC	402	24	0.20	^9,47^
HfN	377 ± 6	17.8 ± 1.1	0.25	^45,46^
HfC	428	25	0.18	^9,47^
VN	393 ± 44	16.0 ± 3.4	0.29	^48^
VC	465	29	0.22	^9,47^
NbN	389 ± 27	25.5 ± 3.7	0.29	^49^
NbC	429	17	0.22	^9,47^
TaN	377 ± 6	30.6 ± 1.3	0.335	^50^
TaC	431	14	0.24	^9,47^
CrC	292 ± 32	17.8 ± 3.8	0.28	^45,46^
CrN	352 ± 31	17.8 ± 2.5	0.29	^45,46^

Results of average nanoindentation hardness and modulus for the high entropy compositions are shown in Table 3; HECN #6 was exempted from mechanical testing due to the two major crystal structures present, which would lead to a multimodal indentation data distribution. Hardness and modulus values for HEN#1 and HECN#1 agree closely with the work of Wen et al.^39^ Compared to literature hardness values for high-entropy carbides^7,9^, HECNs are within the same hardness range, while HENs have lower hardness. All hardness and modulus values are higher than the expected rule-of-mixtures (ROM) of the monocarbide and mononitride constituents for each composition. Nitrides show an average 17% increase in modulus above ROM values and an average 22% increase in hardness above ROM values. Carbonitrides show an average 31% increase in modulus above ROM values and an average 39% increase in hardness above ROM values. ROM hardness and elastic modulus values do not show any trend with actual experimental values, as shown in Supplementary Fig. 3. However, important trends in mechanical properties have arisen with configurational entropy and with valence electron concentration.

**Table 3:** Composition, stoichiometry, density, hardness values, and modulus values for each composition. Theoretical density was calculated using lattice parameters from XRD and atomic ratios from EDS and CHNS chemical analysis, except for HECN#6 which was calculated using the weighted average density of the monocarbide/nitride precursors.

Label	Nominal composition	x (C + N)	Measured density (g/cm^3^)	Theoretical density (g/cm^3^)	Relative density (%)	Nanoindentation hardness (GPa)	ROM hardness (GPa)	Nanoindentation modulus (GPa)	ROM modulus (GPa)	Measured VEC	Single phase?
HEN #1	(HfNbTaTiZr)_1_N_x_	0.88	10.09	10.18	99.1	27.8	22.5	502.6	422.7	8.7	Yes
HEN #2	(CrNbTaTiV)_1_N_x_	0.83	8.33	8.46	98.5	24.4	21.9	476.7	405.0	9.1	Yes
HEN #3	(CrHfNbTaTi)_1_N_x_	0.90	9.50	10.39	91.4	26.2	22.6	488.9	410.7	9.1	Yes
HEN #4	(CrHfNbTiZr)_1_N_x_	0.91	8.15	8.61	94.7	27.3	20.9	488.4	427.4	8.8	Yes
HEN #5	(CrHfTaTiZr)_1_N_x_	0.92	9.56	10.16	94.1	26.5	21.7	465.5	423.9	8.9	Yes
HECN #1	(HfNbTaTiZr)_1_(CN)_x_	0.91	9.54	9.68	98.5	32	22.4	557.9	431.0	8.3	Yes
HECN #2	(CrNbTaTiV)_1_(CN)_x_	0.81	8.16	8.14	100	30	21.9	518.2	415.0	8.6	Yes
HECN #3	(CrHfNbTaTi)_1_(CN)_x_	0.91	10.05	10.21	98.4	29.9	22.0	564.5	419.8	8.7	No—some chemical segregation
HECN #4	(CrHfNbTiZr) _1_(CN)_x_	0.90	8.35	8.43	99.0	30.4	22.5	531.9	429.2	8.4	Yes
HECN #5	(CrHfTaTiZr) _1_(CN)_x_	0.89	9.84	9.86	99.8	29.7	22.5	550	425.8	8.3	Yes
HECN #6	(CrMoTaVW) _1_(CN)_x_	0.65	9.13	10.11	90.3	–	–	–	–	8.3	No—two phases

### Valence electron concentration and hardness

**Figure 4:**
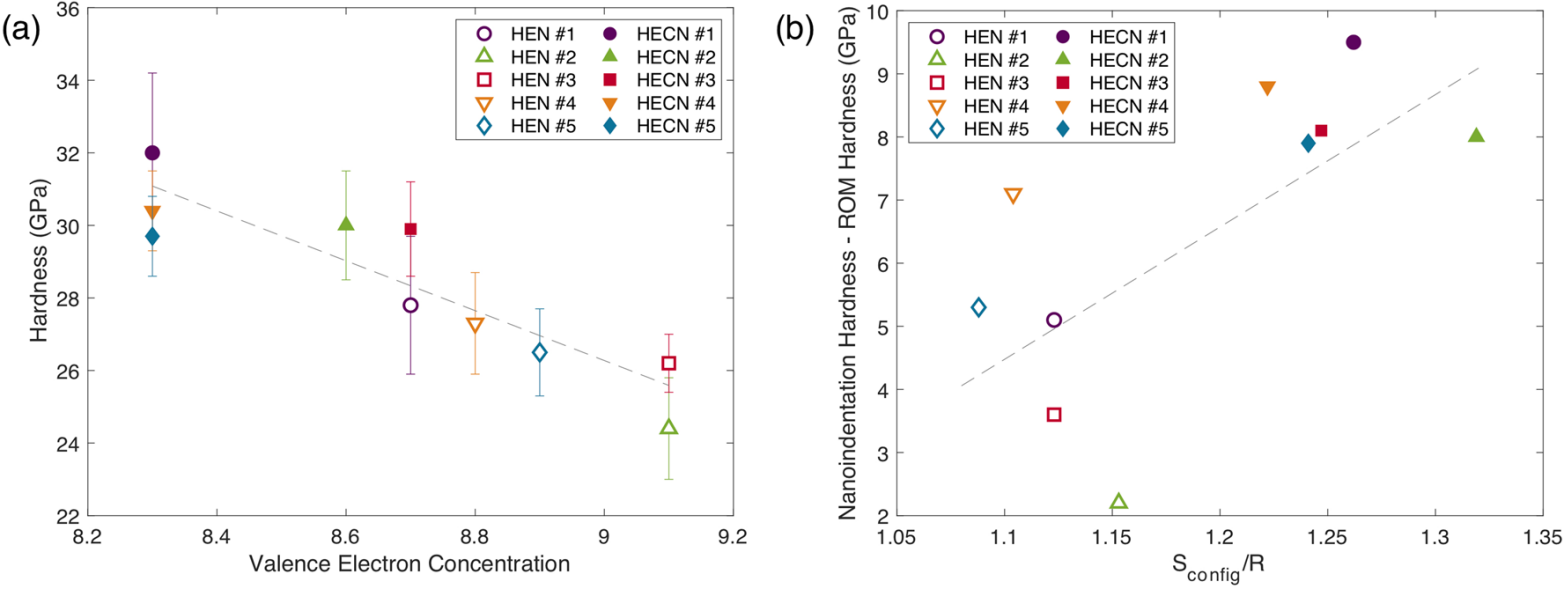
(**a**) Nanoindentation hardness as a function of valence electron concentration (VEC). Error bars are 1 standard deviation from the mean value. (**b**) Increase in hardness as a function of ideal configurational entropy, where R is the gas constant. Pearson correlation coefficients are equal to - 0.90 and 0.75 for (**a**) and (**b**) respectively.

Valence electron concentration (VEC) has been shown to serve as an indicator for mechanical properties in nitrides, carbides, and carbonitrides, as increasing VEC corresponds to increasing metallic bond character and increased number of structural transformations available to the lattice upon deformation^9,40,51^. Here, VEC values are calculated based on the actual measured compositions of each material, and due to vacancies on the anion lattice and chromium loss, this resulted in lower VEC values than expected, by an average of 0.7. In Fig. 4a, nanoindentation hardness has a negative dependence on VEC. Increasing VEC increases metallic bond character and anharmonic lattice vibrations. Therefore, under deformation, the atoms can move to another local minimum structure as opposed to cleaving^41^. Thus, an increase in VEC leads to lower hardness and modulus values. This illustrates the importance of bond character and electronic structure for the intrinsic hardness of these ceramic materials. It is important to keep in mind, however, that VEC, as a way to quantify electronic structure, is an oversimplification, which can be illustrated by the fact that mononitrides with equal VEC can have very different hardness, for example the measured hardness of VN and TaN (VEC = 10) are 16.0 and 30.6 GPa, respectively. However, in high-entropy ceramics, the combined VEC values of the compositions do effectively describe the trends in hardness with composition.

### Randomness, configurational entropy, and hardening

Bulk high-entropy carbides^7,9^ and borides^6^ have shown significantly increased hardness and modulus over their expected ROM values, i.e. the averages of their constituents. In this work, all HENs and HECNs also show increased hardness over ROM values, and, additionally, carbonitrides show an almost double percent increase over the nitrides in both hardness and elastic modulus versus ROM values. To understand why high-entropy materials consistently have higher hardness than their expected values, and further to understand why carbonitrides exhibit higher strengthening than their nitride counterparts, it is necessary to address the compositional randomness in these materials. Wang et al*.*^52^ found that in eight-metal high-entropy carbides, randomness increases hardness due to the increase in possible configurations of dislocation cores, which impedes the movement of dislocations, and that Peierls stress increases with the number of elements. This effect is calculated using density functional theory, where the effect of randomness on specific slip systems can be analyzed. In the same vein, we propose a more facile approach to quantifying the effect of compositional randomness, utilizing configurational entropy.

There are multiple methods used in the literature to calculate ideal configurational entropy of high-entropy materials, with significant inconsistencies; a detailed comparison of these methods is included in the Supplementary Table S3. The crystal structure of high-entropy nitrides and carbonitrides is comprised of two interpenetrating FCC lattices, the metal cation lattice and the anion lattice, with the metals and anions confined to those specific sites. Thus, the configurational entropy is calculated using the sublattice model, which was first described by Temkin in 1945^53^, expanded upon by Hillert^54,55^, and adapted by Miracle and Senkov^56^.

1 $${\Delta {S}_{m}^{ideal}}=\frac{-R\sum_{S}{\sum }_{i}{a}^{S}{X}_{i}^{S}{\mathrm{ln}}\left({X}_{i}^{S}\right)}{{\sum }_{S}{a}^{S}}$$

In Eq. (1), *a*^*s*^ is the number of sites on the *s* sub-lattice, and *X*_*i*_^*s*^ is the fraction of species *i* randomly distributed on the *s* sub-lattice, or the “site fraction.” R is the gas constant, and entropic species (i) are defined as an element^57^ or a point defect^54^. In this version of the sublattice model, dividing by the total number of atom sites (i.e. the number of atoms in the formula unit) results in the configurational entropy on a per mole of atoms basis, so that it is comparable across crystal structures.

For each sample, the experimentally measured composition and vacancy concentration were used to calculate ideal configurational entropy. In the search for the highest-entropy materials, the actual compositional differences from the ideal composition are often overlooked. However, by measuring variations in composition of high-entropy materials, and therefore variations in configurational entropy, the effects of entropy in high-entropy materials can be elucidated. In Fig. 4b, the calculated ideal configurational entropy for each sample is plotted against the increase in hardness over the expected value (calculated as the measured nanoindentation hardness minus the ROM hardness). As configurational entropy increases, the increase in hardness increases. This “entropic strengthening” effect is caused by increased compositional randomness, i.e. the different types and sizes of elements inducing local strain in the lattice which can impede dislocation motion, and it underpins the significant increase in entropy between a five-metal nitride and a five-metal carbonitride.

### Entropic strengthening versus solid solution strengthening

Entropic strengthening explains the hardness increases observed as high-entropy ceramic materials become increasingly complex. The baseline hardness of a high-entropy ceramic material is based on its bonding structure, which can be quantified using VEC, and the increase in hardness over the expected value is based on its increased randomness, or configurational entropy. This is similar to the concept of solid solution strengthening, where the strain fields of atoms of different sizes in a solid solution impede dislocation motion. However, simple measures such as atomic size variance or lattice parameter differences, which capture hardening trends for solid solutions, do not apply to HENs and HECNs in this work, nor HECs in the literature^52^. This is likely due to the fact that each metal atom is coordinated to 6 anions, and vice versa, as opposed to each atom position being equivalent in a solid solution. In fact, Ye et al*.*^58^ have shown that the anion lattice accommodates most of the strain in the lattice in a DFT study on high-entropy carbides. Thus, the assumption in solid solution strengthening that the strain field around each atom position has an effect on the equivalent atom position next to it (i.e. not accounting for different sublattices), causes simple solid solution strengthening parameters to fail.

Conversely, the geometric model for intrinsic residual strain based on atomic size differences developed by Ye et al*.*^59^ does show an interesting correlation with our results. The model was also designed for solid solutions, however, it is based on pairwise geometric comparisons of adjacent atoms, so we adapted the model to only compare the metals with anions in the structure. A limitation of this model is its inability to describe the more complex crystal structures that exist in high-entropy ceramics, including perovskite oxides^14^ and spinels^60^. The root mean squared residual strain calculated with this model correlates extremely well with the calculated configurational entropy values (Pearson correlation coefficient = 0.95, see Supplementary Fig. S6), supporting the notion that configurational entropy is able to capture the intrinsic strain in the lattice caused by compositional randomness. Additionally, entropy’s significance extends further than just size and configuration effects on mechanical properties. Configurational entropy, as a guiding principle of high-entropy materials^61^, already has established implications for phase stability^5^, high-temperature properties^16^, thermal conductivity^62^, and more.

### Stoichiometry

It is worth noting that HEN #2 and HECN #2 fall below the trend for entropic hardening. This can be traced to the fact that those materials have the highest vacancy concentrations of the compositions tested in nanoindentation. Transition metal carbides and nitrides are typically sub-stoichiometric and are stable in a range of stoichiometry from approximately 0.5 < x < 1.0, though in some cases nitrides can be super-stoichiometric (i.e. x > 1.0)^17^. However, vacancies on the anion lattice outweigh vacancies on the metal lattice when nitrides are synthesized at high temperature^43^. Stoichiometries are listed for each composition in Table 3, where x represents the sum of carbon and nitrogen atomic fractions on the anion lattice, calculated by normalizing the metal atomic fraction to 1. Notably, most of the x values are tightly clustered around 0.90, while x = 0.83 for HEN #2 and x = 0.81 for HECN#2. Increasing anion vacancy concentration has been shown to cause lattice relaxation and increase metallic bond character in VN_x_, as there are increased metal–metal orbital interactions in the space left by the vacancy^63,64^. Likewise, in HEN #2 and HECN#2, with drastically higher vacancy concentrations, the material exhibits more metallic character and therefore lower hardness and modulus increases than predicted by the configurational entropy. Across all of the compositions there exists no clear trend in mechanical properties with stoichiometry, as shown in Supplementary Fig. S4. Moreover, the effect of vacancies and resulting increase in metallic bond character demonstrates the need to recognize both VEC/bonding effects and entropic strengthening.

## Discussion

Eleven bulk HENs and HECNs were synthesized for the first time in this study, including 5 single-phase HENs and 4 single-phase HECNs. A single phase high-entropy ceramic is defined based on two essential criteria: a single crystal structure and the homogenous distribution of elements throughout the sample. These two criteria are selected to ensure that there is only one matrix phase in the material, in addition to ensuring the highest degree of homogeneity, randomness, and therefore entropy in the materials. An additional condition to maximize entropy is that the elements on each sublattice should be in equal proportion. The high-entropy nitrides and carbonitrides in this study were all found to have one single FCC rock salt (space group Fm–3m) matrix phase in XRD, except for HECN #6, which contained an FCC rock salt phase and a hexagonal phase. HECN #6 also showed chemical segregation into two distinct phases in EDS. All compositions that were comprised of a single crystal structure also demonstrated chemical homogeneity throughout the high-entropy matrix phase, except for HECN #3, which had islands of minor niobium segregation. Therefore, all compositions besides HECN #6 and HECN #3 are determined to be single-phase high-entropy ceramics.

In addition to maximizing entropy by creating random, homogenous single-phase ceramic materials, entropy is maximized when compositions are equiatomic, so this is often a goal in high-entropy material synthesis. However, by taking into account the experimental variation in composition and vacancy concentrations that occurred during synthesis of multiple HENs and HECNs, it is possible to observe differences in properties due to compositional randomness. To do so, a consistent method for calculating ideal configurational entropy was discussed, which is necessary and long-overdue, due to the foundational nature of configurational entropy to the field of high-entropy materials. Configurational entropy has been used to classify high-entropy alloys and has implications for the mechanical properties and phase stability of these materials. Competing phase formation during synthesis of high-entropy materials with many components is inevitable, and as high-entropy systems increase in ubiquity and complexity, universal adoption of the sublattice model (per mole of atom basis) will bolster our ability to understand and design entropy-related properties.

Hardness enhancements over the rule of mixtures values (measured as percent increase) are almost double for HECNs over HENs. This is attributed to the significant increase in configurational entropy attained by going from a one-anion system (nitride) to a two-anion system (carbonitride). Higher configurational entropy represents local inhomogeneity in lattice structure, which impedes dislocation motion and complicates the available slip systems, thus increasing hardness, and similarly creates local strain in bonds, thus increasing modulus. This local inhomogeneity is a direct result of increased atomic mixing, and therefore is represented by the ideal configurational entropy of the sample, which positively correlates with hardness and modulus—the entropic strengthening effect.

In addition to configurational effects, it is necessary to consider the elemental composition, which dictates the bond character of the materials. Increasing metallic bond character, which is associated with increasing anion vacancy concentration and increasing VEC, is found to have a negative effect on hardness and elastic modulus. The finding of a negative correlation of both hardness and modulus with VEC in bulk HENs and HECNs fits well with both existing computational^40,41,51,65,66^ and experimental^7,9^ results, which demonstrate that bonding and electronic structure govern the inherent mechanical properties in bulk rock-salt structured high-entropy carbides and nitrides. Purposeful design of high-entropy ceramic compositions’ configurational entropy and VEC provides the ability to tune mechanical properties and high temperature phase stability in cubic high-entropy ceramics.

## Supplementary information

Supplementary Information.

## Data Availability

The datasets generated during and/or analyzed during the current study are available from the corresponding author on reasonable request.
