# Supplementary material for: Bulk high-entropy nitrides and carbonitrides
Source: Sci Rep. 2020 Dec 4;10:21288. doi: 10.1038/s41598-020-78175-8 (PMC7718265; doi:10.1038/s41598-020-78175-8)
Supplement: Supplementary file 1 — Supplementary Information. [file 41598_2020_78175_MOESM1_ESM.docx]

Bulk High-Entropy Nitrides and Carbonitrides (Supplementary Information)

Olivia F. Dippo,^1^ Neda Mesgarzadeh,^1^ Tyler J. Harrington,^1^ Grant D. Schrader,^2^ Kenneth S. Vecchio^1,2^

^1^Materials Science and Engineering Program, UC San Diego, La Jolla CA 92037

^2^Department of NanoEngineering, UC San Diego, La Jolla CA 92037

| **Precursor** | **Major Compounds** | | **Minor Compounds (<10%)** | |
| --- | --- | --- | --- | --- |
|  | Stoichiometry | Space group | Stoichiometry | Space group |
| **CrN** | Cr_2_N | P -3 1 m | CrN | F m -3 m |
|  |  |  | Cr_2_O_3_ | R -3 c H |
| **CrC** | Cr_7_C_3_ | P n m a |  |  |
|  | Cr_3_C_2_ | P n m a |  |  |
|  | Cr_23_C_6_ | F m -3 m |  |  |
| **HfN** | Hf | P 63/m m c |  |  |
|  | HfN | F m -3 m |  |  |
| **HfC** | HfC | F m -3 m |  |  |
| **MoC** | Mo_2_C | P b c n |  |  |
| **NbN** | NbN | F m -3 m | NbN | P 63/m m c |
|  |  |  | Nb_4_N_5_ | I 4/m m m |
|  |  |  | Nb_2_O_5_ | P 1 2/m 1 |
| **NbC** | Nb_6_C_5_ | C 1 2/m 1 |  |  |
|  | NbC | F m -3 m |  |  |
| **TaN** | TaN | P -6 |  |  |
| **TaC** | TaC | F m -3 m |  |  |
| **TiN** | TiN | F m -3 m |  |  |
| **TiC** | TiC | F m -3 m |  |  |
| **VN** | VN | F m -3 m | VO_2_ | C 1 2/m 1 |
| **VC** | V_6_C_5_ | P 31 |  |  |
|  | VC | F m -3 m |  |  |
| **WC** | W_2_C | P -3 1 m |  |  |
|  | WC | P -6 m 2 |  |  |
| **ZrN** | ZrN | F m -3 m | ZrO_2_ | P 1 21/c 1 |
| **ZrC** | ZrC | F m -3 m |  |  |

Supplementary Table 1: Phases present in precursor powders used in the synthesis of high-entropy nitrides and carbonitrides, from XRD.

| **Sample** | **Composition** | **Major phase(s)** | **Secondary phase(s) (<5%)** |
| --- | --- | --- | --- |
| HEN #1 | (HfNbTaTiZr)N | (HfNbTaTiZr)N [FCC] | HfO_2_-ZrO_2_ |
| HEN #2 | (CrNbTaTiV)N | (CrNbTaTiV)N [FCC] | Cr_2_O_3_ |
| HEN #3 | (CrHfNbTaTi)N | (CrHfNbTaTi)N [FCC] | Cr_2_O_3_,  HfO_2_ |
| HEN #4 | (CrHfNbTiZr)N | (CrHfNbTiZr)N [FCC] | HfO_2_-ZrO_2,_  Cr_2_O_3_ |
| HEN #5 | (CrHfTaTiZr)N | (CrHfTaTiZr)N [FCC] | HfO_2_-ZrO_2,_  Cr_2_O_3_ |
| HECN #1 | (HfNbTaTiZr)CN | (HfNbTaTiZr)CN [FCC] | HfO_2_-ZrO_2_ |
| HECN #2 | (CrNbTaTiV)CN | (CrNbTaTiV)CN [FCC] | Cr_2_O_3_ |
| HECN #3 | (CrHfNbTaTi)CN | (CrHfNbTaTi)CN [FCC] | Nb-rich carbonitride phase,  Cr_2_O_3_,  HfO_2_ |
| HECN #4 | (CrHfNbTiZr)CN | (CrHfNbTiZr)CN [FCC] | HfO_2_-ZrO_2_ |
| HECN #5 | (CrHfTaTiZr)CN | (CrHfTaTiZr)CN [FCC] | HfO_2_-ZrO_2,_  Cr_2_O_3_ |
| HECN #6 | (CrMoTaVW)CN | Mo_2_C-W_2_C-Cr_2_N [hexagonal],  (CrTaV)CN [FCC] | - |

Supplementary Table 2: Phases present in sintered bulk samples, from XRD (see Figure 2 in main text) and EDS (see Figure 3 in main text and Supplementary Figure 1). All are considered single-phase matrix materials, with most having some second-phase oxides present. HECN #3 is not a single-phase matrix due to the presence of a Nb-rich phase which is not an oxide (from EDS). HECN #6 is not single-phase due to the presence of two major phases, from XRD and EDS.


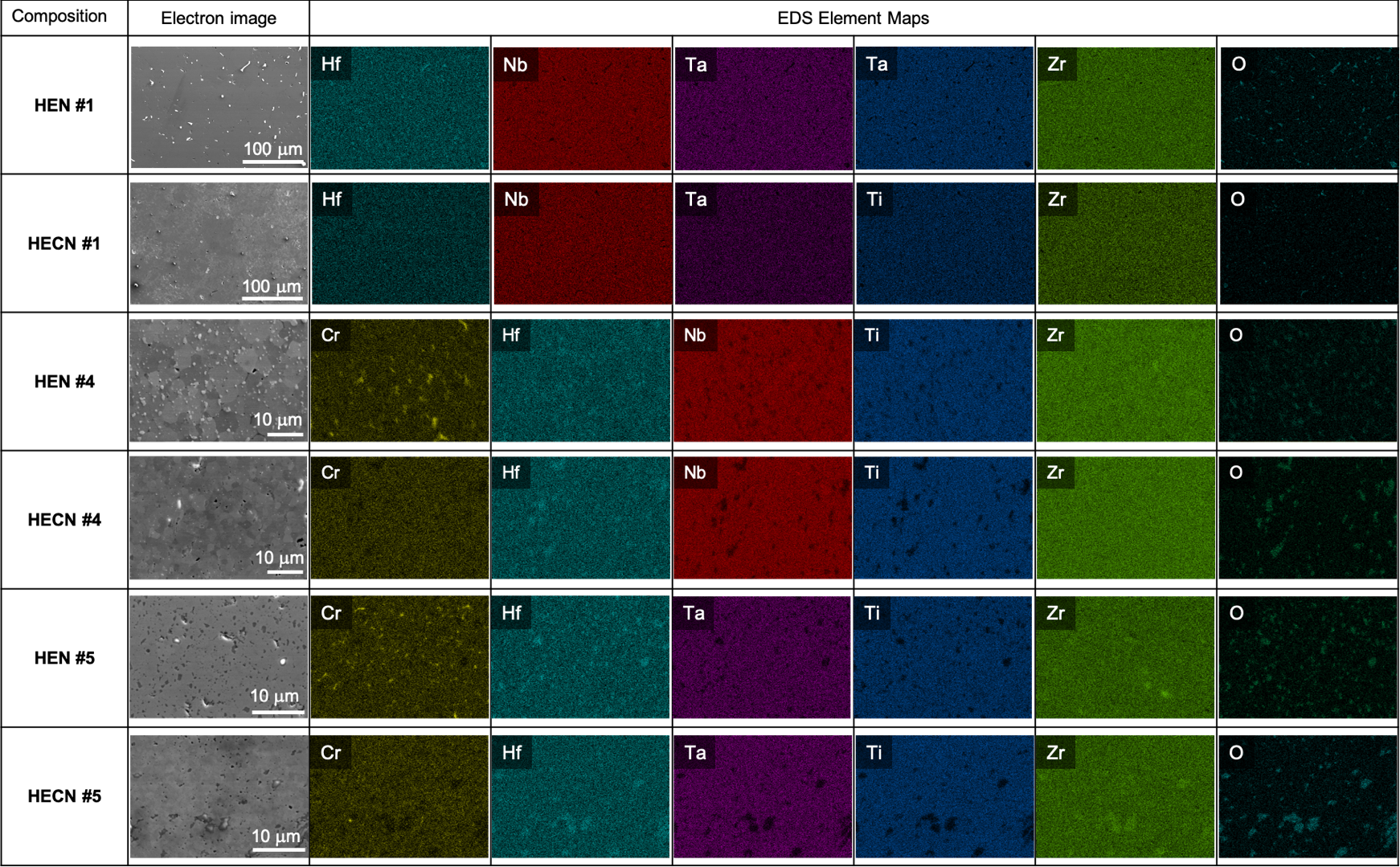
 Supplementary Figure 1: Microstructure and EDS composition maps for HEN and HECN #1,4,5, which are not included in the main text of the paper. Note the different scale in HEN and HECN #1. All compositions shown here are considered single-phase high-entropy compositions. Inhomogeneities seen in the EDS composition maps are minor second-phase oxides that are native to the precursor powders.

Supplementary Figure 2: Nanoindentation hardness as a function of force for composition HECN #2: (CrNbTaTiV)(CN). The minimum force threshold (dotted line) for testing was designated as 100nN. The solid blue line represents an average hardness value of 30.0 GPa from 100 tests at 300 nN. 300nN was determined to be a sufficient load to achieve asymptotic hardness and avoid nanoindentation size effects seen at low loads.

Supplementary Figure 3: Nanoindentation hardness (a) and modulus (b) as a function of the expected rule-of-mixtures (ROM) properties of the binary components. Y=x is displayed as a dashed line. All measured nanoindentation hardness and modulus values are higher than the expected ROM values, though no trend is identified. Error bars are 1 standard deviation from the mean value.

Supplementary Figure 4: Nanoindentation hardness (a) and modulus (b) as a function of anion stoichiometry (carbon+nitrogen). No trend is identified between anion stoichiometry of the high-entropy compositions and mechanical properties. Error bars are 1 standard deviation from the mean value.

|  | **Solid solution model (S.Eq 1)** | **Sublattice model,**  **entropy per mole of formula unit (S.Eq 2)** | **Sublattice model, entropy per mole of atom (S.Eq 3)** |
| --- | --- | --- | --- |
|  | $S_{SS}^{ideal}= -R \sum_{i} X_{i}ln(X_{i})$ | $S_{sub, f.u.}^{ideal}=-R\sum_{S} \sum_{i} a^{S}X_{i}^{S}\ln\left( X_{i}^{S} \right)$ | $\Delta S_{sub}^{ideal}=\frac{-R\sum_{S} \sum_{i} a^{S}X_{i}^{S}\ln\left( X_{i}^{S} \right)}{\sum_{S} a^{S}}$ |
| **(5M)** | ln(5) = 1.61 | ln(5) = 1.61 | ln(5) = 1.61 |
| **(10M)** | ln(10) = 2.30 | ln(10) = 2.30 | ln(10) = 2.30 |
| **(5M)N** | ln(5) = 1.61 | ln(5) = 1.61 | ln(5)/2 = 0.80 |
| **(10M)N** | ln(10) = 2.30 | ln(10) = 2.30 | ln(10)/2 = 1.15 |
| **(5M)(CN)** | ln(10) = 2.30 | ln(5) + ln(2) = 2.30 | [ln(5) + ln(2)]/2 = 1.15 |
| **(5M)_2_(CN)** | ln(10) = 2.30 | 2[ln(5)] + ln(2) = 3.91 | [2(ln(5)) + ln(2)]/2 = 1.30 |

Supplementary Table 3: A comparison of different ways of calculating ideal configurational entropy values for different high-entropy materials. Rows 1 and 2 represent high-entropy alloys with 5 metals (5M) and 10 metals (10M). Rows 3 and 4 represent 5-metal carbonitrides with different stoichiometries. Equation 1 corresponds only to solid solutions (i.e. high-entropy metals with one atom per formula unit). The most commonly used ideal configurational entropy calculation for high-entropy ceramics is Equation 2. However, an inconsistency arises when comparing configurational entropies between phases of different structures. For example, entropy of the 5-metal 2-anion carbonitride has the same entropy as a 10-metal solid solution. That is, the former material, with 7 elements that are restricted in their possible positions to only one of two sublattices, has equal configurational entropy to the latter material which has 10 species that can occupy any position on the lattice. This is fundamentally incorrect, due to the former having fewer possible configurations than the latter, it will have less configurational entropy.

The root of this problem is that the sublattice model in Equation 2 is calculating the configurational entropy *per mole of formula unit*, and where there are unequal numbers of atoms per formula unit (for example, 1 in a solid solution metal, and 2 in a carbonitride), there will be a resulting discrepancy in the configurational entropy. By dividing by the total number of atom sites (i.e. the number of atoms in the formula unit), the configurational entropy is calculated on a per mole of atoms basis, so that it is comparable across crystal structures. As systems increase in complexity, different methods for calculating configurational entropy start to diverge.

Calculating the configurational entropy using the sublattice model per mole of atom (column 3) is the only method that consistently represents the possible number of configurations in the system. the implications of this model include (1) a 5-metal high-entropy nitride has half the ideal configurational entropy of a 5-metal solid solution high-entropy alloy, which is intuitive due to the addition of the ordered carbon sublattice, i.e. only every other lattice position is a random metal in a carbide as opposed to every lattice position in a metal. (2) Differences in stoichiometry are afforded meaningful differences in entropy, as in (5M)(CN) and (5M)_2_(CN). (3) Vacancies and other point defects on each separate lattice can be accounted for as additional species, though this was not specifically shown here. Notably, for this work, the ideal configurational entropy of a 5-metal nitride is 0.8R, and the ideal configurational entropy for a 5-metal carbonitride is 1.15R. A 5-metal 2-anion system has the same ideal configurational entropy value as a 10-metal 1-anion system. That is, by adding one anion to a 5-metal nitride, same entropy gain is achieved as for adding an additional 5 metal species; this is the entropic increase that we exploit by synthesizing carbonitrides, which are 2-anion systems


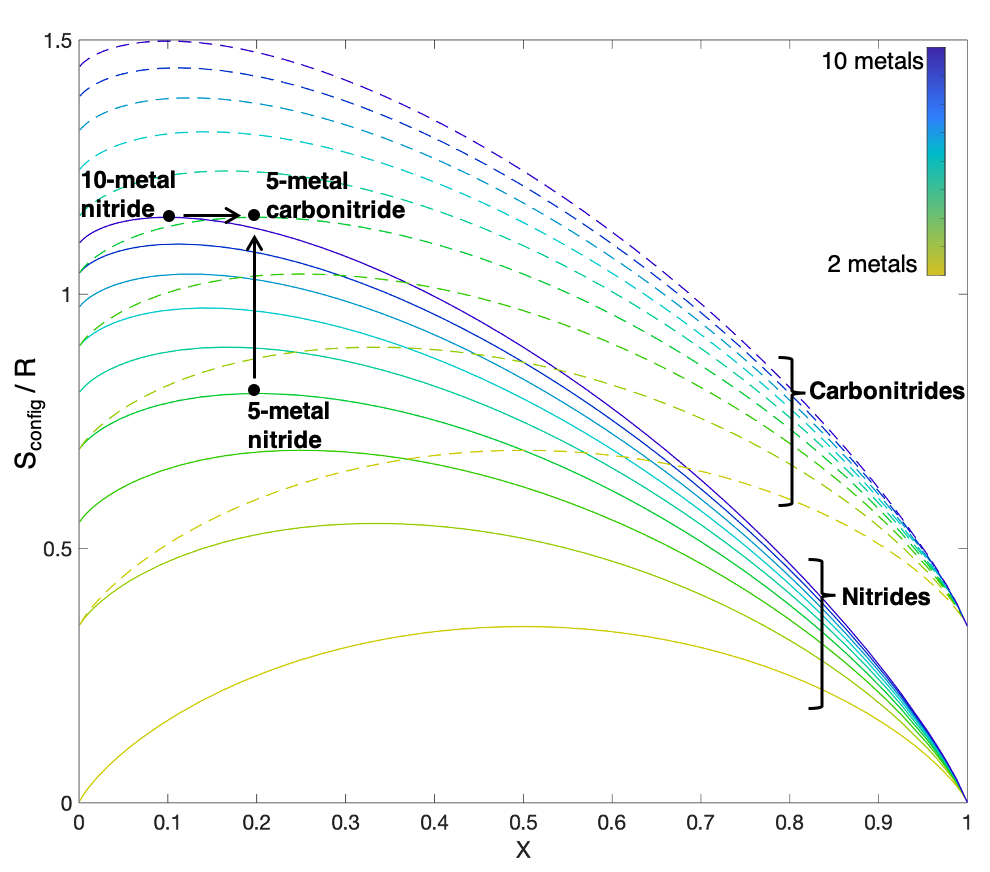


Supplementary Figure 5: The ideal configurational entropy of single anion (i.e. nitride) and two anion (i.e. carbonitride) systems. 10-metal nitrides have equal ideal configurational entropy (S/R) to 5-metal carbonitrides. X corresponds to the site fraction of the metals present in the material, where the maximum entropy corresponds to an equiatomic site fraction.

|  | **Entropy** | **Geometric RMS residual strain** | **Valence electron concentration** |
| --- | --- | --- | --- |
| Hardness | 0.78 | 0.79 | -0.90 |
| Hardness increase | 0.75 | 0.77 | -0.87 |
| Modulus | 0.79 | 0.73 | -0.78 |
| Modulus increase | 0.83 | 0.74 | -0.65 |

Supplementary Table 4: Pearson correlation coefficients for mechanical properties from nanoindentation vs different metrics used to analyze trends. Entropy is calculated using the sublattice model.

Supplementary Figure 6: RMS geometric intrinsic residual strain vs configurational entropy. Dashed line is a linear fit with pearson correlation coefficient = 0.95.

| **Sample** | **Nanoindentation Hardness (GPa)** | **Vickers Hardness (GPa)** |
| --- | --- | --- |
| HEN #1 | 27.8 ± 1.9 | 24.3 ± 1.4 |
| HEN #2 | 24.4 ± 1.4 | 20.0 ± 1.1 |
| HEN #3 | 26.2 ± 0.8 | 22.0 ± 1.5 |
| HEN #4 | 27.3 ± 1.4 | 22.0 ± 2.0 |
| HEN #5 | 26.5 ± 1.2 | 22.8 ± 1.6 |
| HECN #1 | 32.0 ± 2.2 | 30.3 ± 1.6 |
| HECN #2 | 30.0 ± 1.5 | 28.1 ± 1.6 |
| HECN #3 | 29.9 ± 1.3 | 29.1 ± 1.5 |
| HECN #4 | 30.4 ± 1.1 | 28.3 ± 1.2 |
| HECN #5 | 29.7 ± 1.1 | 29.1 ± 2.2 |

Supplementary Table 5: Hardness results from nanoindentation, as described in methods section of main text, and Vickers microhardness indentation, in accordance with ASTM standard C1327, using 100gf load. Vickers hardness results are average of 25 indents, and the error is 1 standard deviation from the mean.

Supplementary Figure 7: Relationship between nanoindentation hardness and Vickers hardness measurements taken from the same materials. Error bars are 1 standard deviation from the mean. Solid gray reference line is y=x.
